# Helical edge states and edge-state transport in strained armchair graphene nanoribbons

**DOI:** 10.1038/s41598-017-08954-3

**Published:** 2017-08-18

**Authors:** Zheng-Fang Liu, Qing-Ping Wu, Ai-Xi Chen, Xian-Bo Xiao, Nian-Hua Liu, Guo-Xing Miao

**Affiliations:** 1 Department of Applied Physics, East China Jiaotong University, Nanchang, 330013 China; 2 Institute for Quantum Computing, University of Waterloo, Waterloo, ON N2L 3G1 Canada; 3 Department of Physics, Zhejiang Sci-Tech University, Hangzhou, 310018 China; 4 School of Computer Science, Jiangxi University of Traditional Chinese Medicine, Nanchang, 330004 China; 5 Institute for Advanced Study, Nanchang University, Nanchang, 330031 China

## Abstract

A helical type edge state, which is generally supported only on graphene with zigzag boundaries, is found to also appear in armchair graphene nanoribbons in the presence of intrinsic spin-orbit coupling and a suitable strain. At a critical strain, there appears a quantum phase transition from a quantum spin Hall state to a trivial insulator state. Further investigation shows that the armchair graphene nanoribbons with intrinsic spin-orbit coupling, Rashba spin-orbit coupling, effective exchange fields and strains also support helical-like edge states with a unique spin texture. In such armchair graphene nanoribbons, the spin directions of the counterpropogating edge states on the same boundary are always opposite to each other, while is not conserved and the spins are canted away from the -direction due to the Rashba spin-orbit coupling, which is different from the case of the zigzag graphene nanoribbons. Moreover, the edge-state energy gap is smaller than that in zigzag graphene nanoribbons, even absent in certain cases.

## Introduction

**Figure 1:**
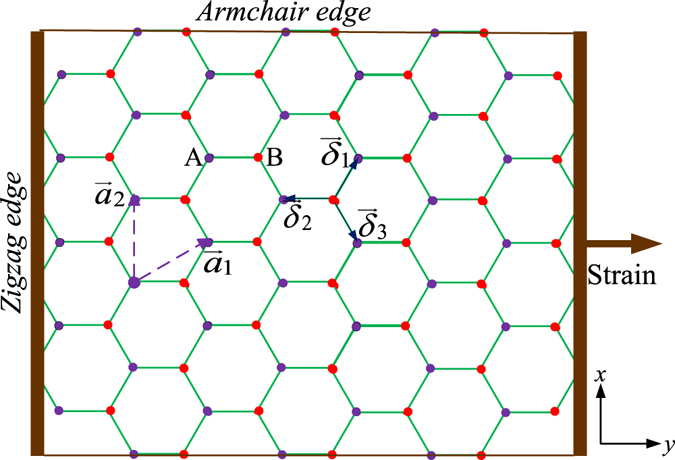
The honeycomb lattice geometry. The lattice structure of graphene is made out of two interpenetrating triangular lattices (**a**_**1**_ and **a**_**2**_ are the lattice unit vectors). The nearest-neighbor vectors are $\mathop{{\delta }_{1}}\limits^{\longrightarrow}$, $\mathop{{\delta }_{2}}\limits^{\longrightarrow}$, and $\mathop{{\delta }_{3}}\limits^{\longrightarrow}$. The armchair edge is at the *x* direction, and Strain is along the *y* direction.

Edge states are special electronic states existing only on the edges, surfaces, or interfaces of materials, and qualitatively distinct from the interior bulk states. They are important to the transport properties especially for low-dimensional systems, because they can provide current carrying channels even when the bulk conductance is gapped, and are often associated with quite distinct and unique properties. For graphene, which is an atomically thin layer of carbon atoms arranged in a honeycomb crystalline lattice^1^, the bulk states follow a linear Dirac dispersion and the bulk carriers are therefore massless and relativistic. On the edges of a graphene sheet, because of the abrupt change in translational symmetry, edge states with different properties appear depending on the type of the edge termination: *zigzag and armchair* (illustrated in Fig. 1, left and top edges). We will focus our attention on functionalizing graphene in order to not only allow charge conductance on these channels, but also permit spin and topology engineering with them.

In the case of zigzag edges, a monolayer graphene naturally supports the dispersionless zero-energy flat bands of edge states^2–4^, present even without magnetic fields^5, 6^. Such flat-band edge states can evolve into helical or chiral ones. In time-reversal (TR) invariant two-dimensional topological insulators, the helical edge state is the key characteristic and naturally gives rise to the quantum spin Hall (QSH) effect^7, 8^. In this QSH phase, current-carrying states are confined on the edge of the sample, whereas the bulk is insulating. These edge states are gapless and protected against backscattering from non-magnetic impurities^9–11^ and their propagation directions are helical, that is, opposite spin states counterpropagate along a given edge of the sample. The QSH state has been observed in HgTe quantum wells^12^, but several works^13–15^ showed that intrinsic spin-orbit coupling (SOC) is probably too small in pristine graphene to allow for experimental verification of this novel phase of matter. Moreover, perturbations violating TR symmetry are usually unavoidable^16^, so people attempt to find QSH-like phases in systems where TR symmetry is broken^16, 17^. The helical edge state can also be induced by other physical origins that may break TR symmetry^18–20^, such as the study on the QSH effect in ferromagnetic graphene^19^.

As for the armchair GNR, edge states can be created in the presence of a magnetic field^21–24^. It is generally believed that without a magnetic field, pristine non-modulated armchair edges cannot support a localized-state band^5, 6, 25, 26^. But people found that modifications on armchair edges can induce complete flat bands, where the wavefunction has the character of valley polarization^27^. The use of edge potentials can also cause the formation of edge states, and the key is to turn on the pseudospin-flipped (intravalley) scattering processes^28^. In this case, the armchair edge bands behave similar to the zigzag ones. However, it has been demonstrated that the properties of armchair localized states depend sensitively on the type of edge modifications^29^.

In addition, it is known that strains can be intentionally introduced on graphene by tensions at the sample-lead contacts on suspended graphene devices^30^, or by deforming the substrates on which the graphene is deposited^31–35^. It was shown that a uniform strain can mimic the effects of a uniform “pseudomagnetic field”, which has opposite signs on the two low-energy graphene valleys^36, 37^ (i.e., in the neighborhoods of the *K* and *K*′ Dirac points). Unlike real magnetic fields, pseudomagnetic fields and strain both preserve the overall TR invariance. Graphene is able to sustain reversible elastic tensile strain of up to 25%^38, 39^. Most importantly, strains can lead to a uniform pseudomagnetic field on the order of 10T in graphene flakes^40^ even 300T in graphene nanobubbles^38^, which can open up interesting applications in graphene nanoelectronics. To date, such pseudomagnetic fields have been realized using two distinct experimental approaches^38, 41^, and in both experiments, the fields realized were strong enough to drive the electronic structures of each valley deep into the quantum Hall regime, in line with theoretical predictions.

In this work, we address one of the crucial features in strained armchair GNRs, namely, the topological nature of their *edge states*. The purpose of this paper is to find a way to tailor the graphene edge states into supporting such nontrivial electronic structures by incorporating a number of intrinsic and extrinsic effects with experimentally realistic parameters. By enabling the armchair boundaries to also support helical edge states, it is possible to realize spin Hall effect all around a piece of graphene independent on its detailed edge structures. The results show that the combined effects of strain and intrinsic SOC can induce spin Hall edge states with nontrivial topology in armchair GNRs, where the spins are split and show a canted helical spin texture in the momentum space.

## System Hamiltonian

We first consider a graphene honeycomb lattice in the *x* − *y* plane in the presence of uniaxial strains with homogeneous Rashba SOC, intrinsic SOC, and an effective exchange fields (EEF) interaction. In this paper, we assume that electrons in the bulk of the graphene sheet are described by the real space *π*-orbital tight-binding Hamiltonian with nearest-neighbor hopping:

1 $$\begin{array}{c}H(r)=\sum _{ < ij > ;\alpha }{t}_{i,j}{c}_{i\alpha }^{\dagger }{c}_{j\alpha }+\frac{2i}{\sqrt{3}}{t}_{SO}\sum _{\langle \langle ij\rangle \rangle }{c}_{i}^{\dagger }{\bf{s}}\cdot {\boldsymbol{(}}{{\bf{d}}}_{kj}\times {{\bf{d}}}_{ik}{\boldsymbol{)}}{c}_{j}\\ \,\,\,\,\,\,\,\,\,\,\,\,\,\,\,+M\sum _{i;\alpha ,\beta }{c}_{i\alpha }^{\dagger }{s}_{\alpha \beta }^{z}{c}_{i\beta }+i{t}_{R}\sum _{ < ij > ;\alpha ,\beta }{{\bf{e}}}_{z}^{0}\cdot ({{\bf{s}}}_{\alpha ,\beta }\times {{\bf{d}}}_{ij}){c}_{i\alpha }^{\dagger }{c}_{j\beta }\mathrm{.}\end{array}$$

Here, ${c}_{i\alpha }^{\dagger }$ and *c*_*iα*_ are *π*-orbital creation and annihilation operators for an electron with spin *α* on site *i*. The first term describes hopping between nearest neighbors *i, j* on the honeycomb lattice with the hopping amplitude ${t}_{ij}={t}_{i}={t}_{0}{e}^{-\mathrm{3.37(}{\delta }_{i}-\mathrm{1)}}$ ^42^, where the unstrained graphene hopping amplitude is ${t}_{0}\approx -2.9{\rm{eV}}$, the deformed and undeformed lattice distances are *δ*_*i*_ and *a*_0_. Because the tension is along the armchair direction, the deformed bond lengths $|{\delta }_{1}|=|{\delta }_{3}|\mathrm{=1}+\frac{3}{4}\varepsilon -\frac{1}{4}\varepsilon \sigma$, $|{\delta }_{2}|\mathrm{=1}-\varepsilon \sigma$, where *σ* = 0.165 is the Poisson’s ratio of graphite and *ε* is the tensile strain^43^. The second term is the mirror symmetric intrinsic SOC with a coupling strength *t*_*SO*_. Here ${\bf{s}}=({s}^{x},{s}^{y},{s}^{z})$ are the Pauli matrices, and *i* and *j* refer to the next-nearest neighboring sites that have a common nearest neighbor *k* connected by vectors **d**_*ik*_ and **d**_*kj*_. **d**_*ij*_ represents a unit vector pointing from site *j* to site *i*. The third term corresponds to a uniform out-of-plane EEF, and *M* is the exchange field strength. The last term represents the Rashba SOC with coupling strength *t*_*R*_.

In the geometry of Fig. 1, the unit-cell vectors of the undeformed lattice are ${{a}}_{1}=\frac{{a}_{0}}{2}(\sqrt{3},\mathrm{3)}$, ${{a}}_{2}=\frac{{a}_{0}}{2}\mathrm{(2}\sqrt{3},\mathrm{0)}$, and the corresponding reciprocal-lattice vectors are given by ${{b}}_{1}=\frac{2\pi }{{a}_{0}}(\mathrm{0,}\,\frac{2}{3}),{{b}}_{2}=\frac{2\pi }{{a}_{0}}(\frac{1}{\sqrt{3}},\,\frac{-1}{3})$. Two Dirac points are situated at particular points ${K}=\frac{2\pi }{{a}_{0}}(\frac{2}{3\sqrt{3}},\,0)$ and ${K}\text{'}=\frac{2\pi }{{a}_{0}}(\frac{-2}{3\sqrt{3}},\,0)$. In the deformed lattice, modification to these distances distorts the reciprocal lattice as well, and positions of the high-symmetry points are also shifted. For uniaxial tension along the armchair direction, the two Dirac points move to the new positions ${K}=\frac{4\pi }{3\sqrt{3}{a}_{0}}(1-\frac{\varepsilon \mathrm{(1}-\sigma )}{2},\,0)$ and ${K}\text{'}=-\frac{4\pi }{3\sqrt{3}{a}_{0}}(1-\frac{\varepsilon \mathrm{(1}-\sigma )}{2},\,0)$. With the increase of tension, the Dirac points always approach each other and will eventually merge. Moreover, the system can become gapped at a critical tensile strain $\varepsilon  > 0.23$ ^42^, which means a phase transition is triggered here.

Unless many-body effects are of crucial importance, in the vicinity of Dirac points *K* or *K*′, the low-energy electronic properties of a monolayer graphene are well described by the Dirac-type equation

2 $$H=-i\hslash {\nu }_{F}(\tau {\sigma }_{x}{k}_{x}+{\sigma }_{y}{k}_{y}){{\bf{1}}}_{s}+\tau {\lambda }_{SO}(x){\sigma }_{z}{s}_{z}+M{{\bf{1}}}_{\sigma }{s}_{z}+\tau {\bf{A}}\cdot {\sigma }_{i}\mathrm{.}$$

here *σ*_*i*_ and *s*_*i*_ $(i=x,y,z)$ are the Pauli matrices acting on the sublattice (*A*,*B*) and physical spin ($\uparrow ,\downarrow$) spaces, respectively. *τ* = ± labels *K* and *K*′ valleys. The Fermi velocity and intrinsic SOC are given by ${\nu }_{F}=\frac{3{a}_{0}t}{2}$ and ${\lambda }_{SO}=3\sqrt{3}{t}_{SO}$. In most of the following expressions we set $\hslash {\nu }_{F}=1$ for simplicity. ${\bf{A}}=A(x,y)$ is the in-plane pseudogauge field induced by the uniaxial strain, which is defined as^36, 44–46^

$$(\begin{array}{c}{A}_{x}\\ {A}_{y}\end{array})=t\beta (\begin{array}{c}-2{u}_{xy}\\ {u}_{xx}-{u}_{yy}\end{array})$$

where *u*_*ij*_ is the in-plane strain tensor. The constant $\beta =\partial \,\mathrm{ln}\,t/\partial \,\mathrm{ln}\,\delta$, where *t* is the nearest-neighbour hopping parameter, and *δ* is the distance between nearest carbon atoms^47, 48^. Due to the *y* axis is oriented along the armchair direction, ${u}_{xx}=-\varepsilon \sigma$ and ${u}_{yy}=\varepsilon$; as a result, the strain causes only a finite but constant ${A}_{y}=A=t\beta \varepsilon$ $\mathrm{(1}+\sigma )$ ^46^ while *A*_*x*_ = 0. This can be taken into account by simply shifting *P*_*y*_ in this region. The wave function corresponds to a spinor comprising four components ${{\rm{\Psi }}}_{K(K^{\prime} )}(x,y)=({{\rm{\Psi }}}_{A\uparrow K(K^{\prime} )},{{\rm{\Psi }}}_{B\downarrow K(K^{\prime} )},{{\rm{\Psi }}}_{B\uparrow K(K^{\prime} )},{{\rm{\Psi }}}_{A\downarrow K(K^{\prime} )})$.

### Electronic states of Armchair GNRs within the continuum Dirac model

In this section, we calculate the electronic states of an armchair GNR within the continuum Dirac model. To understand the role of strains clearly, we only consider the effects of intrinsic SOC and strains in this section. Dispersion relation of the armchair GNR can be derived by solving the Schröinger equation in the following form

3 $$H(\begin{array}{c}{{\rm{\Psi }}}_{A\uparrow K(K^{\prime} )}\\ {{\rm{\Psi }}}_{B\downarrow K(K^{\prime} )}\\ {{\rm{\Psi }}}_{B\uparrow K(K^{\prime} )}\\ {{\rm{\Psi }}}_{A\downarrow K(K^{\prime} )}\end{array})=E(\begin{array}{c}{{\rm{\Psi }}}_{A\uparrow K(K^{\prime} )}\\ {{\rm{\Psi }}}_{B\downarrow K(K^{\prime} )}\\ {{\rm{\Psi }}}_{B\uparrow K(K^{\prime} )}\\ {{\rm{\Psi }}}_{A\downarrow K(K^{\prime} )}\end{array}),$$

here the Hamiltonian can be expressed as:

$$H=[\begin{array}{cccc}\tau {\lambda }_{SO} & 0 & -{\partial }_{y}-\tau i({\partial }_{x}+A) & 0\\ 0 & \tau {\lambda }_{SO} & 0 & {\partial }_{y}-\tau i({\partial }_{x}-A)\\ {\partial }_{y}-\tau i({\partial }_{x}-A) & 0 & -\tau {\lambda }_{SO} & 0\\ 0 & -{\partial }_{y}-\tau i({\partial }_{x}+A) & 0 & -\tau {\lambda }_{SO}\end{array}]\mathrm{.}$$

where ${{\rm{\Psi }}}_{A(B)\alpha K(K^{\prime} )}$ is the wave function of spin $\alpha (\uparrow ,\downarrow )$ state on the sublattice *A*(*B*) near the *K* and *K*′ points.

Translational symmetry guarantees that the total wave function can be written in the form

$$(\begin{array}{c}{{\rm{\Psi }}}_{A\uparrow K(K^{\prime} )}\\ {{\rm{\Psi }}}_{B\downarrow K(K^{\prime} )}\\ {{\rm{\Psi }}}_{B\uparrow K(K^{\prime} )}\\ {{\rm{\Psi }}}_{A\downarrow K(K^{\prime} )}\end{array})={e}^{i{k}_{y}y}(\begin{array}{c}{\varphi }_{A\uparrow K(K^{\prime} )}\\ {\varphi }_{B\downarrow K(K^{\prime} )}\\ {\varphi }_{B\uparrow K(K^{\prime} )}\\ {\varphi }_{A\downarrow K(K^{\prime} )}\end{array})\mathrm{.}$$

Upon solving Eq. (3), we obtain the energy dispersion relation:

4 $$E=\mu \sqrt{{({k}_{y}+\tau A)}^{2}+{k}_{x}^{2}+{\lambda }_{SO}^{2}}\mathrm{.}$$

where *μ* = ± stands for the conduction (+) and valence (−) bands.

The general solution of Eq. (3) is a sum of plane waves

5 $$\{\begin{array}{c}{\varphi }_{A\uparrow (\downarrow )K(K^{\prime} )}={a}_{A\uparrow (\downarrow )K(K^{\prime} )}{e}^{i{k}_{x}x}+{b}_{A\uparrow (\downarrow )K(K^{\prime} )}{e}^{-i{k}_{x}x}\\ {\varphi }_{B\uparrow (\downarrow )K(K^{\prime} )}={c}_{B\uparrow (\downarrow )K(K^{\prime} )}{e}^{i{k}_{x}x}+{d}_{B\uparrow (\downarrow )K(K^{\prime} )}{e}^{-i{k}_{x}x}\end{array}\mathrm{.}$$

The total wave function has the form ${\rm{\Psi }}={e}^{iK\cdot {\bf{r}}}{{\rm{\Psi }}}_{K}+{e}^{iK\text{'}\cdot {\bf{r}}}{{\rm{\Psi }}}_{K^{\prime} }$.

Due to the boundary conditions at the edges of the ribbon (located at *x* = 0 and *x* = *L*, where *L* is the ribbon width),

Using the above equations, we can obtain:

6 $$\{\begin{array}{rcl}{\varphi }_{A\uparrow K(K^{\prime} )} & = & \tau {k}_{x}-i({k}_{y}+A)]{e}^{i\tau {k}_{x}x}\\ {\varphi }_{B\uparrow K(K^{\prime} )} & = & \tau (E-{\lambda }_{SO}){e}^{i\tau {k}_{x}x}\\ {\varphi }_{A\downarrow K(K^{\prime} )} & = & \tau (E-{\lambda }_{SO}){e}^{i\tau {k}_{x}x}\\ {\varphi }_{B\downarrow K(K^{\prime} )} & = & \tau {k}_{x}+i({k}_{y}+A)]{e}^{i\tau {k}_{x}x}.\end{array}$$

**Figure 2:**
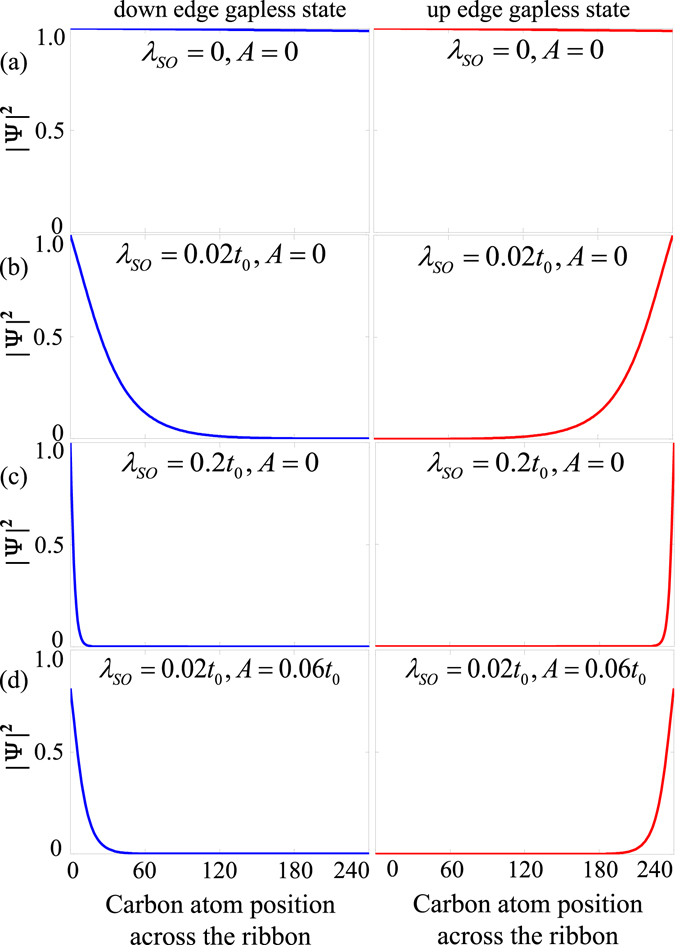
Probability density across the single layer armchair GNR of the edge states in the presence of different intrinsic SOC and strain ${\lambda }_{so}=0$ and *A* = 0 (**a**), ${\lambda }_{so}=0$ and *A* = 0 (**b**), ${\lambda }_{so}=0.2{t}_{0}$ and *A* = 0 (**c**), and ${\lambda }_{so}=0.02{t}_{0}$ and $A=0.06{t}_{0}$ (**d**). The GNR width is set to be 240 carbon atoms.

Where, ${k}_{x}=\sqrt{{E}^{2}-{({k}_{y}+A)}^{2}-{\lambda }_{SO}^{2}}$. If *k*_*x*_ are real, Eq. (6) corresponds to confined modes in the graphene ribbon; while imaginary *k*_*x*_ corresponds to edge states because they decay exponentially into the ribbon. But the intrinsically small *λ*_*SO*_ makes the edge states negligibly small in general. Fortunately, Eq. (6) shows that the dispersion of the wave function may be modulated with strains. In Fig. 2, we plot the probability density profile of the edge state wave functions. The evolution of the edge states with increasing intrinsic SOC and strains are clearly shown: the edge states are absent in the unmodulated armchair GNR [Fig. 2(a)]; even for ${\lambda }_{SO}=0.02{t}_{0}$, the probability density still mainly shows the characteristics of bulklike states (these electronic edge states not only transport along the edges of graphene but also penetrate into the interior of the system obviously) [Fig. 2(b)] akin to that of armchair graphene nanoribbons modulated by edge potential^28^; only for large intrinsic SOC [Fig. 2(c)], the armchair GNR can embody obvious features of edge states, which is however difficult to achieve experimentally. But a striking finding is that, when strains are also considered (e.g. $A=0.06{t}_{0}$, that is, $\varepsilon \approx 0.03$), the armchair GNR can support the edge state structures even for small intrinsic SOC [Fig. 2(d)]. Here the role of strains is not to change the energy gap but to modulate the phase position. To understand the behavior of this kind of edge states more clearly, we further use the tight-binding method to study the edge states of the armchair GNRs in the presence of intrinsic SOC, Rashba SOC, EEF, and strains in the next section.

### Edge states and transport properties of Armchair GNRs

It is well known that intrinsic SOC can induce helical edge states in GNRs with zigzag edges^7, 8^. Generally, such helical edge states are absent in armchair GNRs. If armchair GNRs could also support helical edge states, we would obtain a way to achieve QSH effect independent of the graphene’s edge structures. In addition, strains can also induce QH effect^41^, so we analyse the edge state structure of an armchair GNR with the modulation of intrinsic SOC and strains by calculating the band energy, conductance and local density of states (LDOS). The conductance and LDOS are calculated with the non-equilibrium Green’s functions (NEGF). Across the whole system, the conductance from an arbitrary lead *l* to lead *r* is given by ref. 49

7 $$G={e}^{2}/hTr[{{\rm{\Gamma }}}_{l}{G}_{D}{{\rm{\Gamma }}}_{r}{G}_{D}^{\dagger }],$$

and

8 $${G}_{D}={[E-{H}_{D}-{{\rm{\Sigma }}}_{r}^{R}-{{\rm{\Sigma }}}_{l}^{R}]}^{-1},{{\rm{\Gamma }}}_{l,r}=i({{\rm{\Sigma }}}_{l,r}^{R}-{{\rm{\Sigma }}}_{l,r}^{R\dagger }\mathrm{).}$$

Here ${G}_{D}({G}_{D}^{\dagger })$ is the device retarded(advanced) Green’s function, ${{\rm{\Gamma }}}_{l,r}$ is the broadening function, and ${{\rm{\Sigma }}}_{l,r}^{R}$ is the boundary self energy term. The diagonal elements of the total Green’s function yield the LDOS at the site $r$ $\rho (r,E)=-\frac{1}{\pi }Im[{G}_{D}(r,r,E)]$.

**Figure 3:**
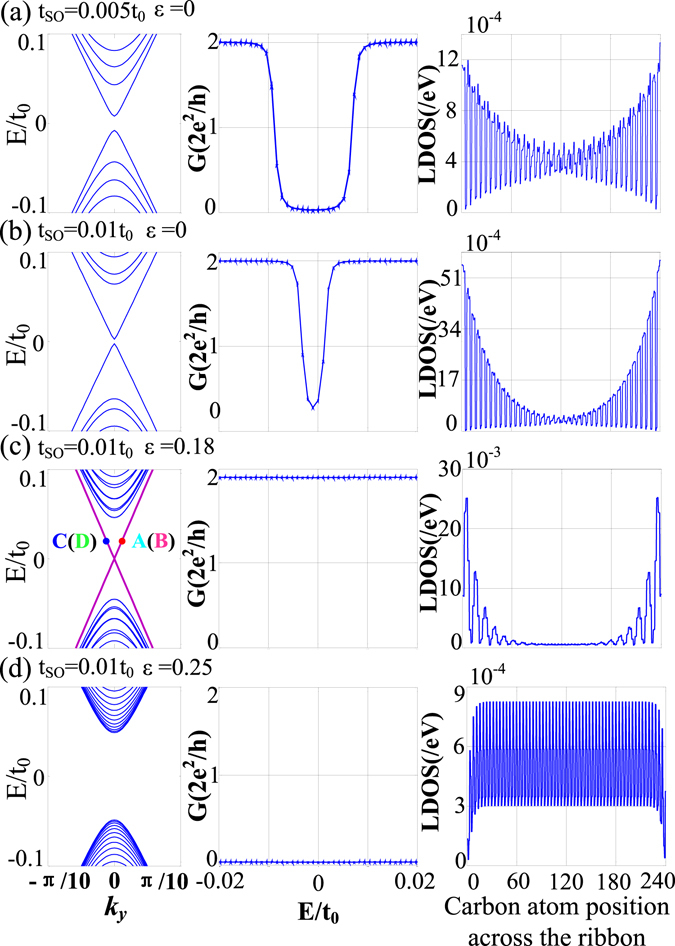
Edge-state band structure, conductivity *G* and LDOS of armchair GNR for $\{{t}_{so}=0.005{t}_{0},\varepsilon =0\}$ (**a**), $\{{t}_{so}=0.01{t}_{0},\varepsilon =0\}$ (**b**), $\{{t}_{so}=0.01{t}_{0},\varepsilon =0.18\}$ (**c**), and $\{{t}_{so}=0.01{t}_{0},\varepsilon =0.25\}$ (**d**) in the tight-binding model. The GNR width is set to be 240 carbon atoms.

Firstly, we only consider the role of intrinsic SOC on the energy band structures of an armchair GNR [Fig. 3(a)–(b)]. Figure 3(a) shows that there is an obvious edge-state band gap in the energy spectrum for small intrinsic SOC, which can also be seen from the edge-state conductance in Fig. 3(a). Such an energy band structure results in the absence of edge states, which can also be seen from the LDOS [Fig. 3(a)]. Even for ${t}_{SO}=0.01{t}_{0}$ [Fig. 3(b)], the LDOS still embody the features of bulk states. The edge-state band gap still exists but negligibly small. As a result, the edge-state conductance is nonzero due to tunneling effect. When the strain is also considered, the edge-state band gap disappears [Fig. 3(c)], and we find that the LDOS distribution has the typical characteristics of edge state structures, which makes the edge-state conductance nonzero and constant. Therefore strains are very important to edge-state transport. But it is well known that large strains can also generate a bulk spectral gap for $\varepsilon  > {\varepsilon }_{C}\approx 0.23$ ^42^. Such a bulk spectral gap makes the above edge-state features disappear for $\varepsilon =0.25$ [Fig. 3(d)]. As a result, the edge-state conductance *G* = 0 and the LDOS resembles the features of bulk states and is completely suppressed on the edges.

The above discussions show that strains can assist the intrinsic SOC in generating edge states in the armchair GNR. Next, we analyze the probability density of the edge-state wavefunctions using the tight-binding method. As to be shown below, GNRs with armchair edges, when modulated with intrinsic SOC and strains, can possess robust gapless helical edge states akin to those from zigzag GNRs modulated with only intrinsic SOC.

**Figure 4:**
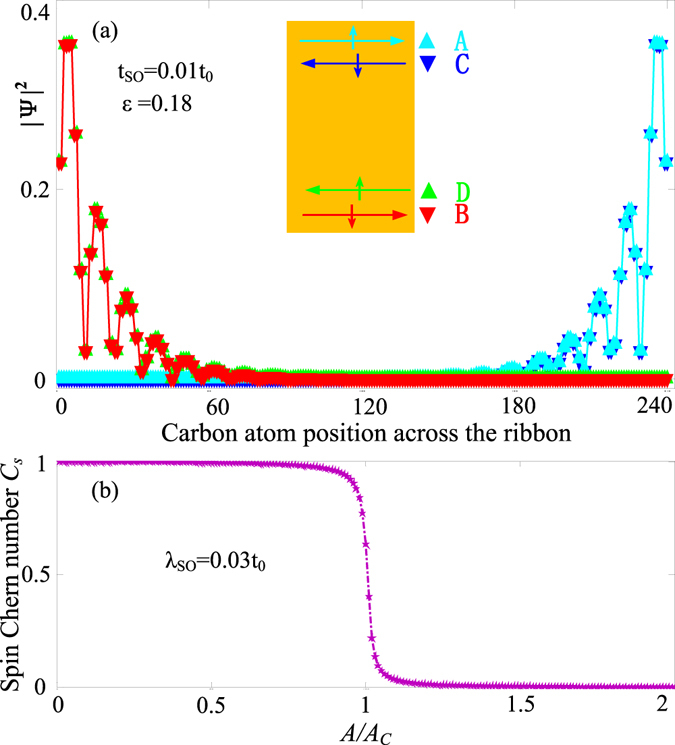
(**a**) Probability densities of the wave functions of the edge states labeled by A, B, C, D in Fig. 3(c), the inset is the schematics which indicate the propagation directions of the edge modes. (**b**) The calculated spin Chern numbers within the continuum Dirac model as a function of strain $A$ with ${\lambda }_{so}=0.03{t}_{0}$. The GNR width is set to be 240 carbon atoms.

The seminal work of Kane and Mele^50^ showed that intrinsic SOC can open a topologically nontrivial gap in the zigzag GNR at zero magnetic field. This bulk gap hosts two counterpropagating edge modes per edge with opposite spins (one Kramers pair), which are the helical edge states related by TRS. This topological phase is known as the QSH state, and may be regarded as two opposite QH phases (i.e., each spin produces one copy of QH effect, with opposite chirality). The TR invariance is considered a prerequisite for the QSH effect. As discussed above, the combined effect of the strain and the intrinsic SOC not only induces the edge states, but also preserves the overall TR invariance^36^. Now we further consider the probability density of these edge-state wavefunctions [Fig. 4(a)]. For a given Fermi level in the gap [as marked in Fig. 3(c)], there exist four edge states labeled as **A**, **B**, **C**, and **D**, note that **A** and **B** (**C** and **D**) are degenerate. From ${\bf{v}}({\bf{k}})=\frac{1}{\hslash }\frac{\partial E({\bf{k}})}{\partial {\bf{k}}}$, one can find that states **A** and **B** (**C** and **D**) propagate along the same *y*(−*y*) direction. By analyzing the probability density of the edge-state wavefunctions for the states labeled as **A**, **B**, **C**, and **D** in Fig. 3(c), one can find that the wave functions of state **A** with spin up and positive velocity and state **C** with spin down and negative velocity are localized on the upper boundary, whereas state **B** with spin down and positive velocity and state **D** with spin up and negative velocity counterpropagate along the lower boundary [Fig. 4(a)]. This indicates that the system possesses the desired QSH features with a helical edge state structure.

The emergence of helical edge states in the bulk gap is intimately related to the topological properties of the bulk Bloch states in the valence bands, which can be described by the *Z*_2_ indices^50^ or the spin Chern numbers^17, 51, 52^. It has been shown that the *Z*_2_ indices and spin Chern numbers yield equivalent descriptions for TR invariant systems^52–56^. Here we utilize the spin Chern numbers to describe the characters of the QSH effect in graphene with intrinsic SOC and strains. The spin Chern number is defined as ${C}_{s}=\frac{1}{2}{\sum }_{\alpha \beta }\alpha {C}_{\alpha \beta }$, here ${C}_{\alpha \beta }$ is the Chern number for the spin-*αβ* sector and can be expressed as ${C}_{\alpha \beta }={\sum }_{\eta }{C}_{\alpha \beta }^{\eta }$, $\eta =\pm$ is the valley index. The valley Chern number can be calculated from

9 $${C}_{\alpha \beta }^{\eta }=\frac{1}{2\pi }\sum _{n}{\int }_{BZ}d{k}_{x}d{k}_{y}{({{\rm{\Omega }}}_{xy}^{n})}_{\alpha \beta }^{\eta },$$

here $\alpha (\beta )=\pm$ is the spin index and ${{\rm{\Omega }}}_{xy}^{n}$ is the Berry curvature of the $n$th band

10 $${{\rm{\Omega }}}_{xy}^{n}={\partial }_{{k}_{x}}{A}_{{k}_{y}}-{\partial }_{{k}_{y}}{A}_{{k}_{x}},$$

${A}_{{k}_{x}({k}_{y})}$ is the Berry connection, which is defined as

11 $${A}_{{k}_{x}({k}_{y})}=\sum _{{\rm{occupied\; state}}}\langle {\rm{\Phi }}|i{\partial }_{{k}_{x}({k}_{y})}|{\rm{\Phi }}\rangle ,$$

where ${\rm{\Phi }}$ represents the eigenstates of the Hamiltonian defined in Eq. (2) and can be written as:

12 $${\rm{\Phi }}=(\begin{array}{c}{{\rm{\Phi }}}_{A\uparrow K(K^{\prime} )}\\ {{\rm{\Phi }}}_{B\uparrow K(K^{\prime} )}\\ {{\rm{\Phi }}}_{A\downarrow K(K^{\prime} )}\\ {{\rm{\Phi }}}_{B\downarrow K(K^{\prime} )}\end{array})=(\begin{array}{c}\tau {k}_{x}-i({k}_{y}+\tau A)\\ {E}_{k(k^{\prime} )}-\tau {\lambda }_{SO}\\ {E}_{k(k^{\prime} )}-\tau {\lambda }_{SO}\\ \tau {k}_{x}+i({k}_{y}+\tau A)\end{array})\mathrm{.}$$

Where ${E}_{k}=-\sqrt{{({k}_{y}+A)}^{2}+{k}_{x}^{2}+{\lambda }_{SO}^{2}}$ and ${E}_{k}^{\text{'}}=-\sqrt{{({k}_{y}-A)}^{2}+{k}_{x}^{2}+{\lambda }_{SO}^{2}}$. Figure 4(b) plots the spin Chern number as a function of the strain for ${\lambda }_{SO}\,=\,0.03{t}_{0}$. In Fig. 4(b), we observe that there is a critical strain value ${A}_{C}=\hslash {\nu }_{F}{k}_{y}^{m}$, where ${k}_{y}^{m}$ is the maximal value of *k*_*y*_ in the first Brillouin zone, and if the strain exceeds the critical value *A*_*C*_, the spin Chern number ${C}_{s}\approx 0$, in line with the edge-state conductance in Fig. 3(d). On the other hand, for $A < {A}_{C}$, the spin Chern number ${C}_{s}=1$, in agreement with the number of helical edge states from the tight-binding calculations, i.e., the QSH effect can be formed in the armchair GNR with suitable strains. Note that the spin Chern number is defined by Eq. (12) without considering any boundary conditions^57^. One can see that the spin Chern number is still $1$ under the condition *A* = 0, which can be achieved in zigzag GNRs. Here we would like to emphasize that strains not only can induce the QSH effect in the armchair GNR, but also can achieve a quantum phase transition from the QSH phase to a trivial insulator phase.

**Figure 5:**
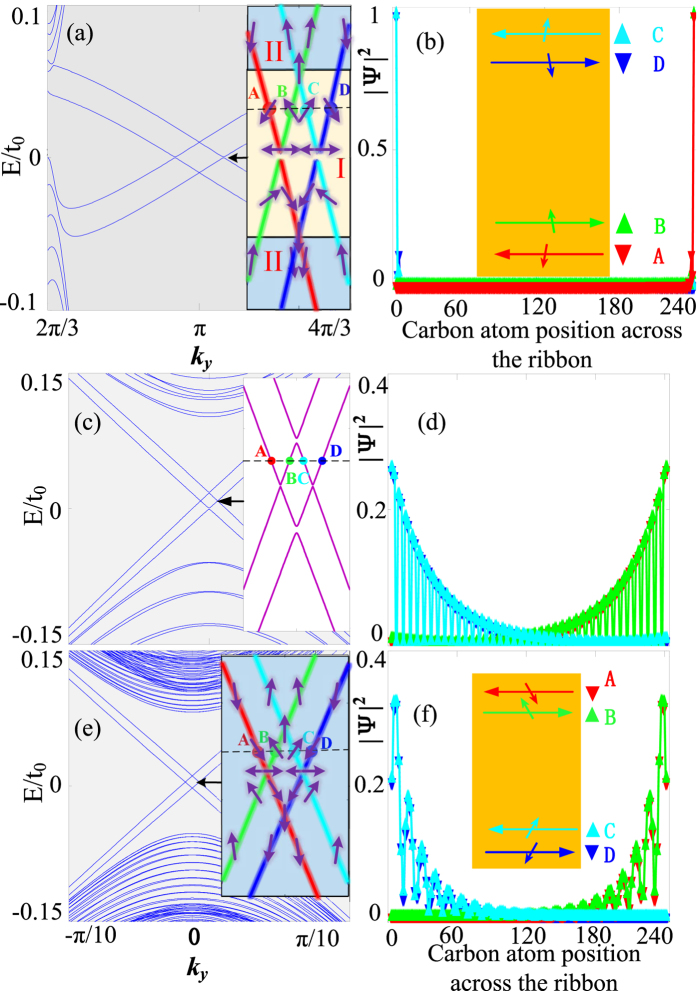
Edge-state band structures and probability densities of the edge-state wave functions of zigzag (**a**,**b**) and armchair (**c**–**f**) GNRs for $\{{t}_{so}=0.01{t}_{0},{t}_{R}=0.02{t}_{0},M=0.01{t}_{0}\}$. The strain is set as *ε* = 0 for (**a**–**d**); and *ε* = 0.18 for the other figures. The GNR width is set to be 240 carbon atoms.

Usually, the QSH state of matter is considered to be protected by the TR symmetry. But QSH-like phases in systems where the TR symmetry is broken have also been suggested^17^. It was found that the QSH-like state appears in a zigzag GNR with the combined effects of intrinsic SOC, Rashba spin-orbit coupling and an exchange field. Here we further consider the effect of strains on such QSH-like states in a zigzag GNR. From Fig. 5(a), one can easily distinguish the edge states from the bulk states. Due to the Rashba SOC, *s*_*z*_ is not conserved and the spins of the edge states can be rotated to canted orientations. Therefore, we compute the spin polarization ${P}_{m}^{j}=\langle m|{\sum }_{i}{c}_{i}^{\dagger }{s}_{j}{c}_{i}|m\rangle$ (where $|m\rangle$ is the *m*th band, *i* and *j* are the lattice site indices, and $j=x,y,z$) to obtain information about the spin states, and then label the spin directions with the small arrows in Fig. 5(a). Through analysis on the spatial distribution of the wave functions, we find that the spin-textured edge states can be divided into two regions at low energies, and regardless of the spin-texture regions, the edge states from the bands labeled with red and green (blue and cyan) lines are mainly located on the lower (upper) boundary. However, the spin textures are significantly different in these two regions. For region I [shaded yellow in Fig. 5(a), close to the Dirac points], spins of the counterpropogating states on the same boundary are not strictly opposite to each other, only their out-of-plane components are opposite. In Fig. 5(b), we plot the probability density profile of edge state wave functions for the four edge states labeled with **A**, **B**, **C** and **D** in Fig. 5(a), and we find that edge states with average spin down and negative velocity (red symbols) and those with average spin up and positive velocity (green symbols) propagate along the lower boundary, and edge states with average spin down and positive velocity (blue symbols) and those with average spin up and negative velocity (cyan symbols) propogate along the upper boundary, in agreement with ref. 17. The case of region II(further away from the Dirac points) is different from that of region I. In region II, the spin directions of the counterpropogating states on the same boundary are nearly opposite to each other. Thereby, varying the GNR’s chemical potentials also can fine tune the spin directions in the edge conductance channels.

We further investigate the fate of the QSH-like effect in the armchair GNR in the presence of intrinsic SOC, Rashba SOC, strains and an exchange field. In the absence of strains, the edge states show obvious features of bulk states, although there exist edge states in the bulk gap [Fig. 5(c)–(d)]. If the strain is also considered, one can find clear edge states appearing in the bulk gap [Fig. 5(e)], and the edge states from the bands labeled with red and green (blue and cyan) lines are located mainly on the upper (lower) boundary. Moreover, the probability density profile of the edge-state wave functions of the armchair GNR is similar to that of the zigzag GNR [Fig. 5(f)], that is to say, the armchair GNR in the presence of intrinsic SOC, Rashba SOC, strains and EEF also supports the QSH-like edge states. But there are distinct differences between the edge states of Fig. 5(b) and (f). In Fig. 5(b), the counterpropogating spins on the same boundary are not strictly opposite to each other. As a comparison, for the armchair GNR, the counterpropogating spins on the same boundary are strictly opposite to each other [Fig. 5(f)]. Such differences can be attributed to their different spin textures. For the zigzag GNR, its spin texture is divided into the two regions as described above [Fig. 5(a)], with spins in region I significantly noncollinear. Yet in the armchair GNR, the spin directions of the edge states on the same boundary are strictly opposite to each other, which is robust under the low energy conditions. From Fig. 5(a), one can also note that there is a small energy gap in the edge-state spectrum, leading to a low-dissipation spin transport similar to the result of ref. 17. But in the armchair GNR with the same modulations, such energy gap is smaller and even absent [Fig. 5(e)], which can greatly weaken scattering between forward and backward movers^17^, realizing a dissipationless spin transport.

## Summary

In this paper, we studied the edge-state properties of armchair GNRs with intrinsic SOC, Rashba SOC, EEF and strains. Within the continuum Dirac model, we predicted that the armchair GNR, when strained along the armchair direction, can also support edge states even for very small intrinsic SOC. The strains can to some extent compensate the weak intrinsic SOC in graphene. Moreover, the conductance and LDOS, calculated based on the NEGF, show that the edge-state conductivity is quantized. And analysis of the spatial distribution of the wave functions and spin Chern numbers further illustrates that such edge states are helical type edge states, which confirms the presence of spin Hall effect in the armchair GNR. The spin Chern number further shows that a quantum phase transition from a QSH state to a trivial insulator state happens at a critical strain.

Owing to the broken *s*_*z*_ spin conservation induced by the Rashba SOC, the spin textures are obviously different between the armchair GNR and the zigzag GNR. Specifically, in the zigzag GNR, the spin texture is closely related to the Fermi energy. For energies close to the Dirac point, the edge-state spin directions are not strictly opposite to each other for the counterpropogating states on the same boundary, only their out-of-plane components are opposite. And for energies further away, the spin directions of these states on the same boundary are opposite to each other. As a comparison, in the armchair GNR, the spin texture is invariant to the Fermi energy and the spin directions of these states on the same boundary are always opposite to each other. In addition, we also observe that a small edge-state band gap appears for the zigzag GNR, while for the armchair GNR, the edge-state energy gap is smaller and even absent.

In summary, helical dege states are supported by not only the zigzag GNR but also the armchair GNR when strains are present, and the strained armchair GNR can have unique advantage in achieving QSH-like effects with broken TR symmetry.
